# Supplementary material for: Serum Response Factor-Regulated IDO1/Kyn-Ahr Pathway Promotes Tumorigenesis of Oral Squamous Cell Carcinoma
Source: Cancers (Basel). 2023 Feb 19;15(4):1319. doi: 10.3390/cancers15041319 (PMC9954402; doi:10.3390/cancers15041319)
Supplement: Supplementary file 1 [file cancers-15-01319-s001.zip › cancers-2194606-supplementary.pdf]

Serum Response Factor-Regulated IDO1/Kyn-Ahr Pathway Promotes Tumorigenesis of Oral Squamous Cell Carcinoma

Mingyan Xu, Feixiang Zhu, Qi Yin, Hao Yin, Shaobin Fang, Gongwei Luo, Jie Huang, Wenxia Huang, Fan Liu, Ming Zhong, and Xiaoling Deng

Supplementary Figures

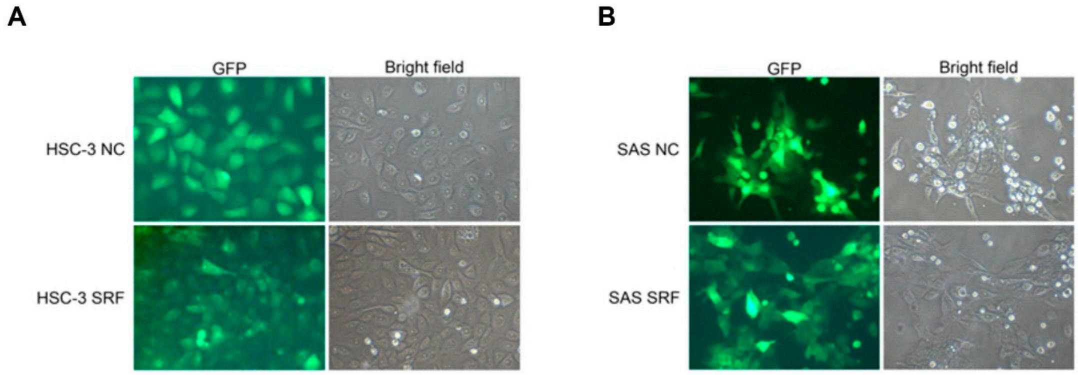

Supplementary Figure S1. Construction and validation of stable cell lines overexpressing SRF. Fluorescence detection of HSC-3 (A) and SAS (B) cells after stably transfected with lentivirus containing pCDH-GFP or pCDH-h-SRF-GFP. GFP, green fluorescent protein; SRF, serum response factor.

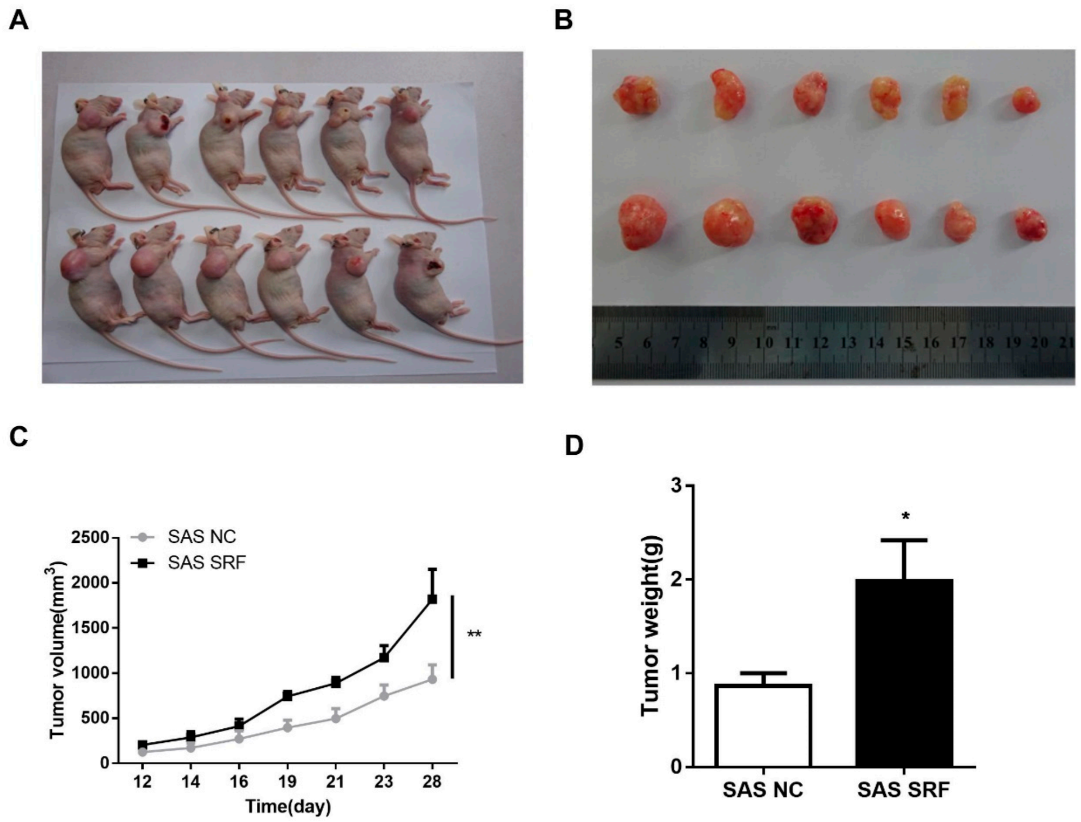

Supplementary Figure S2. SRF overexpression promotes SAS cell tumorigenesis *in vivo*.

(A and B) Images of nude mice and tumors isolated from a xenograft mouse model. 2×10^5 SAS cells infected with the indicated lentiviruses were resuspended in PBS and then injected into nude mice (n= 6/group). Tumors were removed from nude mice euthanized 28 days later. (C) Tumor volumes were measured with calipers on the indicated days (**$p < 0.01$ vs SAS NC). (D) The weights of dissected xenograft tumors were significantly increased in mice overexpressing SRF (*$p < 0.05$ vs SAS NC). Results from three independent measurements are shown as means ± SEMs. SRF, serum response factor.

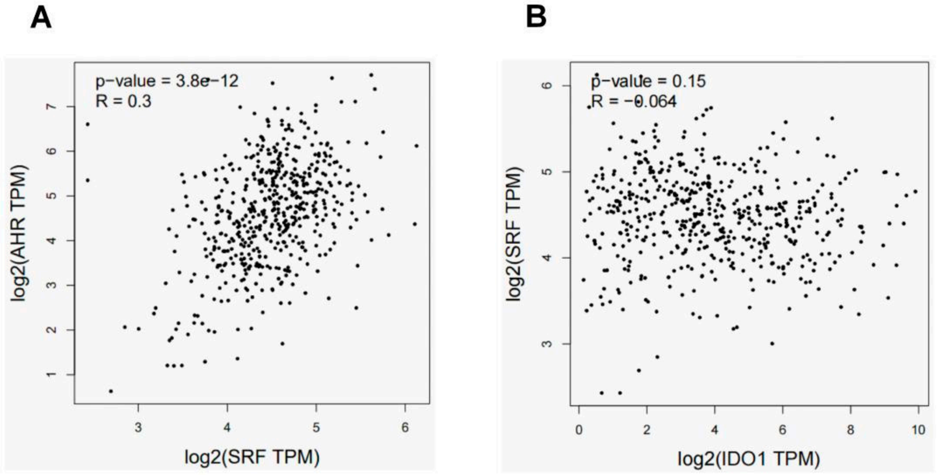

Supplementary Figure S3. Association between *SRF* and *AhR* or *IDO1* mRNA expression in head and neck tumors based on the Cancer Genome Atlas (TCGA) database. (A) *SRF* mRNA expression was positively correlated with *AhR* mRNA expression in head and neck squamous cell carcinoma (HNSCC) based on TCGA database. (B). Association of *SRF* and *IDO1* mRNA expression was not statistically significant for HNSCC in TCGA database. SRF, serum response factor.

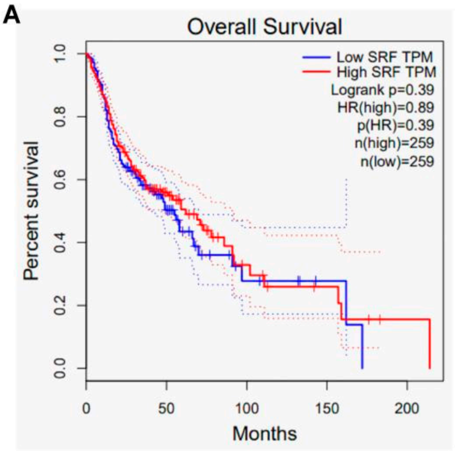

Supplementary Figure S4. Overall survival analysis for HNSCC patient and *SRF* mRNA expression in TCGA database. Kaplan-Meier survival curve for overall survival time of HNSCC patients and *SRF* mRNA expression in TCGA database (p>0.05). SRF, serum response factor.

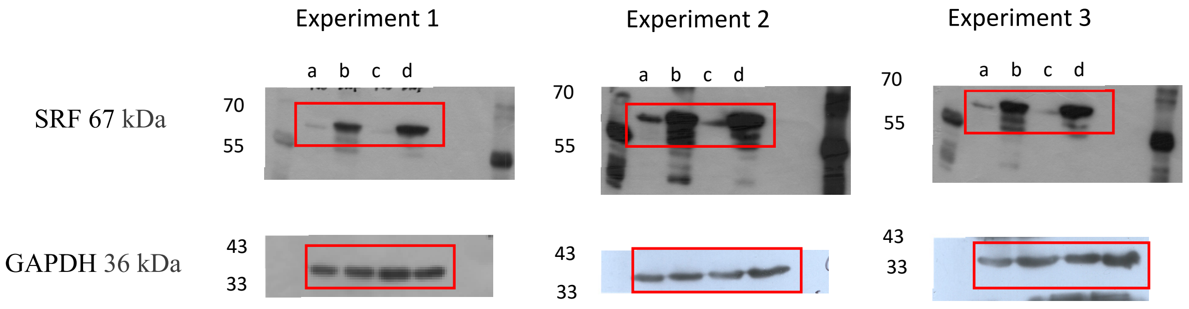

Supplementary Figure S5. Western blotting image source. Original Western blotting images displayed in Figure 2B. Experiment 1 images were displayed in the main figure. a, SAS-Ctrl; b, SAS-pCGN-SRF; c, HSC-3-Ctrl; d, HSC-3-pCGN-SRF.

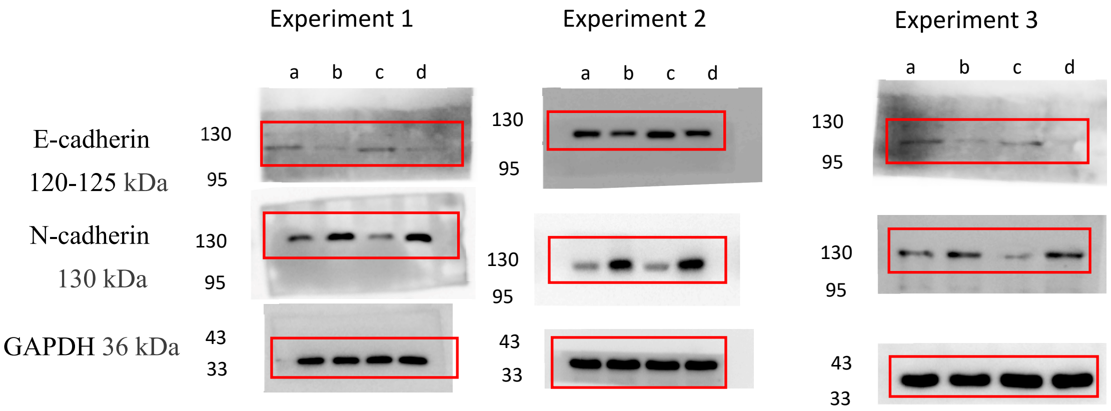

Supplementary Figure S6. Western blotting image source. Original Western blotting images displayed in Figure 3A. Experiment 2 images were displayed in the main figure. a, SAS-NC; b, SAS-SRF; c, HSC-3-NC; d, HSC-3- SRF.

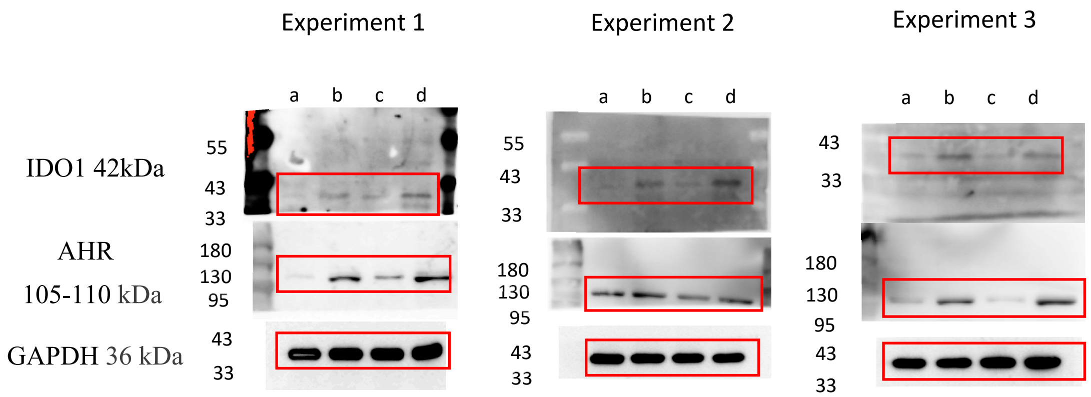

Supplementary Figure S7. Western blotting image source. Original Western blotting images displayed in Figure 5F. Experiment 1 images were displayed in the main figure. a, SAS-NC; b, SAS-SRF; c, HSC-3-NC; d, HSC-3- SRF.

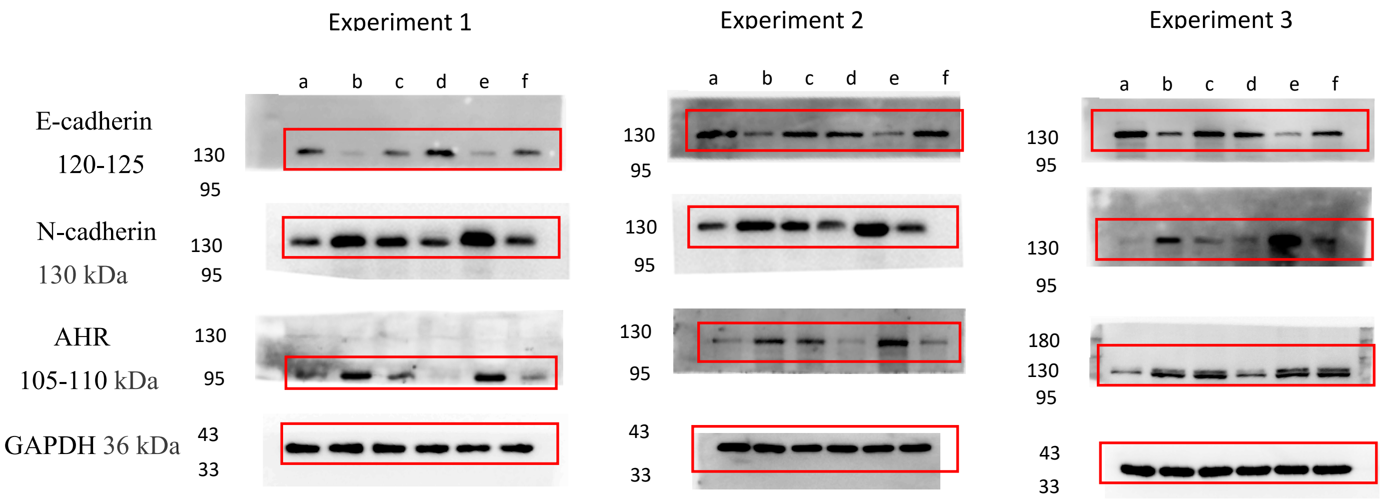

Supplementary Figure S8. Western blotting image source. Original Western blotting images displayed in Figure 7E. Experiment 1 images were displayed in the main figure. a, SAS-NC; b, SAS-SRF; c, SAS-SRF+PC; d, HSC-3-NC; e, HSC-3-SRF; f, HSC-3-SRF+PC.

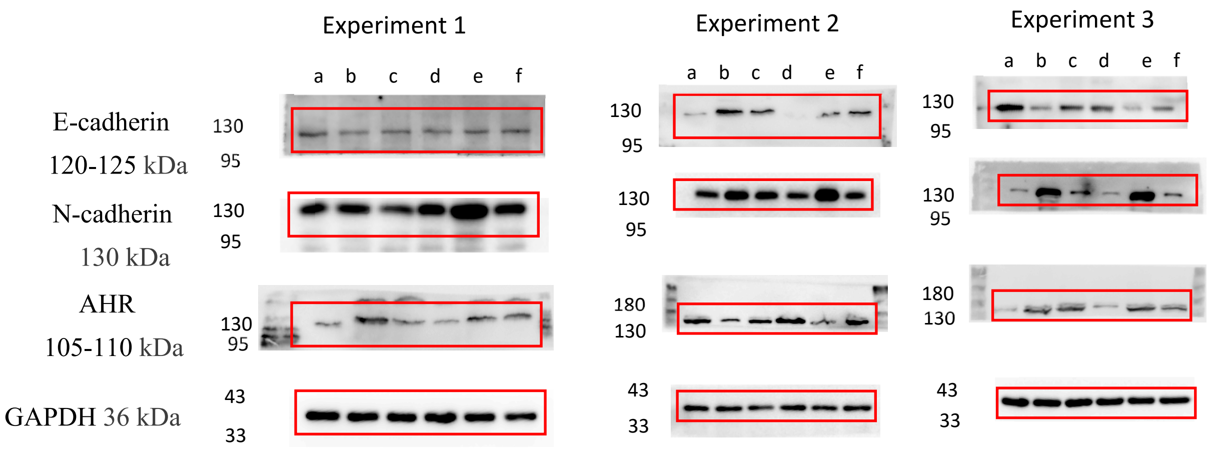

Supplementary Figure S9. Western blotting image source. Original Western blotting images displayed in Figure 8E. Experiment 3 images were displayed in the main figure. a, SAS-NC; b, SAS-SRF; c, SAS-SRF+PDM2; d, HSC-3-NC; e, HSC-3-SRF; f, HSC-3-SRF+PDM2.
